# A genome-wide association study for reading and language abilities in two population cohorts

**DOI:** 10.1111/gbb.12053

**Published:** 2013-06-20

**Authors:** M Luciano, D M Evans, N K Hansell, S E Medland, G W Montgomery, N G Martin, M J Wright, T C Bates

**Affiliations:** † Centre for Cognitive Aging and Cognitive Epidemiology, Department of Psychology, University of EdinburghEdinburgh; ‡ MRC Centre for Causal Analyses in Translational Epidemiology, School of Social and Community Medicine, University of BristolBristol, UK; § Genetic Epidemiology, Queensland Institute of Medical ResearchBrisbane, Australia

**Keywords:** Association, dyslexia, genes, GWAS, language, reading

## Abstract

Candidate genes have been identified for both reading and language, but most of the heritable variance in these traits remains unexplained. Here, we report a genome-wide association meta-analysis of two large cohorts: population samples of Australian twins and siblings aged 12–25 years (*n* = 1177 from 538 families), and a younger cohort of children of the UK Avon Longitudinal Study of Parents and their Children (aged 8 and 9 years; maximum *n* = 5472). Suggestive association was indicated for reading measures and non-word repetition (NWR), with the greatest support found for single nucleotide polymorphisms (SNPs) in the pseudogene, *ABCC13* (*P* = 7.34 × 10^−8^), and the gene, *DAZAP1* (*P* = 1.32 × 10^−6^). Gene-based analyses showed significant association (*P* < 2.8 × 10^−6^) for reading and spelling with genes *CD2L1*, *CDC2L2* and *RCAN3* in two loci on chromosome 1. Some support was found for the same SNPs having effects on both reading skill and NWR, which is compatible with behavior genetic evidence for influences of reading acquisition on phonological-task performance. The results implicate novel candidates for study in additional cohorts for reading and language abilities.

Reading and language disorders are heritable, with genetic effects accounting for between 45% and 61% of the phenotypic variance (Bishop *et al*. 2006; Harlaar *et al*. 2005; Hawke *et al*. 2006). Moreover, dyslexia and specific language impairment (SLI) are often comorbid, with evidence of shared genetic etiology (Bishop 2001; Bishop & Hayiou-Thomas 2008; Newbury *et al*. 2011; Rice *et al*. 2009). The identification of putative genes influencing reading disability has been relatively successful compared with other complex cognitive traits. Linkage analysis identified a number of chromosomes of interest (e.g. 3, 6p, 15q) and fine mapping of these regions implicated strong candidate genes—including *ROBO1* (Hannula-Jouppi *et al*. 2005), *DCDC2* (Meng *et al*. 2005), *KIAA0319* (Cope *et al*. 2005) and *DYX1C1* (Taipale *et al*. 2003)—both linkage and gene associations have been replicated, although null replications have also been reported (e.g. Skiba *et al*. 2011; Tran *et al*. 2013; Zhong *et al*. 2013; Zou *et al*. 2012). For SLI, evidence from linkage and targeted association studies has converged on two regions of interest, 7q and 16 (SLI Consortium 2002), and three candidate genes, *CNTNAP2*, *CMIP* and *ATP2C2* (Newbury *et al*. 2009; Vernes *et al*. 2008), with evidence suggesting that these genes exert pleiotropic effects on language and reading (Newbury *et al*. 2011). The genetic linkage and association findings for dyslexia and language impairment may also generalize to normal variation in reading and language (Bates *et al*. 2010; Lind *et al*. 2010; Luciano *et al*. 2007; Whitehouse *et al*. 2011). Here, we report a genome-wide association study (GWAS) for these traits in individuals from population-based samples.

One GWAS for reading ability has previously been reported. This used a multistage DNA-pooling design in which the allele frequencies of low and high reading ability groups were compared using a 100K single nucleotide polymorphism (SNP) microarray (Meaburn *et al*. 2008). A subsequent design stage involved individual genotyping and confirmed support for association with 10 SNPs; although these SNPs were not replicated in an independent cohort (Luciano *et al*. 2011). Field *et al*. (2013) carried out genome-wide association (on only 133 165 SNPs) in the context of a family design (718 individuals from 101 families with dyslexia probands) but found no significant associations. More recently, an event-related brain potential phenotype, the mismatch negativity, has been subjected to GWAS in a sample of individuals with dyslexia, and showed replicable association with rs4234898, a marker located on chromosome 4q32.1 and associated with mRNA-expression levels of *SLC2A3*, a neural glucose transporter gene (Roeske *et al*. 2011). No GWAS of the non-word repetition (NWR) marker of SLI risk has been reported to date to our knowledge. The present GWAS meta-analysis of reading traits capturing phonological decoding and orthographic skill and of NWR performance—a marker of SLI—is undertaken in a primarily adolescent sample from the general Australian population and a UK population sample of children. It, then, is the first GWAS of these traits using continuously varying reading and language measures from the general population and comprehensive GWAS data, that is, imputed to ∼2.4 million SNPs.

## Materials and methods

### Sample

#### Brisbane Adolescent Twin Sample (BATS)

Twins and their non-twin siblings were recruited from ongoing studies of melanoma risk factors and cognition in a population-based sample (Wright *et al*. 2001). This including 1177 individuals from 538 families (134 monozygotic twins, 338 dizygotic twins, two families comprising both a monozygotic and dizygotic twin pair, 11 triplets, three dizygotic pairs + one or two siblings, six pairs of non-twin siblings, one set of three siblings and 43 individuals—unpaired twins/non-twins) who had Components of Reading Examination (CORE) reading (described below), intelligence quotient (IQ) and genotyping data. A subset including 1111 individuals from 505 families had language, IQ and genotyping data, and 1057 individuals from 479 families had whole word reading, IQ and genotyping data. For monozygotic twin pairs the mean of their test scores was used to increase power (Miller 1998). The age range of the sample was between 12.3 and 25.1 years [mean = 17.9, standard deviation (SD) = 2.9] at the time of testing reading, and ranged 13.7–26.1 years (mean = 20.1, SD = 3.4) at the time of collecting language data. The sample was 54.5% female, and 98% reported Caucasian ancestry, predominantly Anglo-Celtic (∼82%). Written informed consent was obtained from each participant and their parent/guardian (if younger than 18 years). Ethical approval for this study was received from the Human Research Ethics Committee, Queensland Institute of Medical Research.

#### *Avon Longitudinal Study of Parents and their Children* (ALSPAC)

Participants formed part of the ALSPAC, a longitudinal, population-based sample recruited from the county of Avon, UK in the early 1990s (see Golding *et al*. 2001). In this study, phenotype and genotype data were available for the following number of participants: 5078 (reading at age 9), 5070 (non-word reading at age 9), 5071 (spelling at age 9) and 5472 (NWR at age 8). Their IQ was measured at age 8. Written informed consent was obtained from parents and ethical approval was granted from the ALSPAC Law and Ethics committee and other local ethics committees. Participants in both cohorts were free of neurological conditions and major psychiatric illness.

### Measures

#### BATS

Regular-word, irregular-word and non-word reading and spelling were assessed using the CORE (Bates *et al*. 2004), which was lengthened to a 120-word version (Castles & Coltheart 1993) to include spelling and to increase the difficulty level for an older sample. This test was administered over the telephone by a trained researcher. Test scores for the individual component subtests were calculated as a simple sum of correct items and were Box-Cox transformed to attain normality, and a principal component from these tests derived representing a general factor of reading and spelling ability. The reading and spelling factor was assessed to tap general processes underlying reading and spelling abilities. A measure of whole word reading, the Schonell graded word reading test (Schonell & Schonell 1960), which tested irregular and regular words, was also investigated. The Schonell reading data were negatively skewed and transformed by a logarithmic function of the reverse distribution. The assessment of language ability (representing phonological storage efficiency) was via the Gathercole and Baddeley (Gathercole 1994) and Dollaghan and Campbell (Dollaghan & Campbell 1998) NWR tests; the sum of the standard scores from each measure was used to increase trait reliability. Intelligence [performance IQ from the Multidimensional Aptitude Battery (Jackson 1984)] was entered as a covariate to increase the sensitivity to detect genetic effects for reading ability, as has been previously shown (Luciano *et al*. 2007), and language skill independent of IQ. This has the effect of adjusting reading and language scores that are low because of low general cognitive ability. The IQ and the Schonell were measured as close as possible to participants’ 16th birthday. The CORE measures were collected between 1 month and 8.3 years before IQ in 53.8% of the sample, with the remainder being collected between 1 month and 9 years after IQ administration. The NWR and CORE measures were collected at the same time in 29.6% of the sample, with the remaining sample being assessed on NWR up to 4.1 years after CORE assessment.

#### ALSPAC sample

Reading was assessed using separate tests of word reading, non-word reading and spelling. Reading items were chosen from a larger selection of words used by Nunes *et al*. (2003). Test–retest reliability of word reading was 0.80 and had a correlation of 0.85 with the Schonell Graded Word Reading Task and 0.81 with the word spelling test given 4 months later. Test–retest reliability of the non-word reading task was 0.73 and showed a correlation of 0.73 and 0.77 with the reading and spelling tasks, respectively, given 4 months later. Language was assessed by an adapted NWR test comprised of 12 non-words, four each of three, four and five syllables and conforming to phonological rules for sound combinations. Each word was presented via a cassette recording and the participant was required to repeat each item, with a correct score given if there was no phonological deviation from the target item. A composite measure of word reading, non-word reading and spelling was formed, providing a measure comparable to the reading and spelling factor assessed in the BATS cohort. The IQ was measured with items based on the Wechsler tests, in this case a short form of the WISC-III (Wechsler *et al*. 1992) in which alternate items were used for all subtests, except the coding subtest which was administered in full. The performance IQ measure was used.

### Genotyping, quality control and imputation

#### BATS

DNA was extracted from blood samples and SNP genotyping was performed with the Illumina 610k Quad Bead chip by deCODE Genetics (Reykjavik, Iceland). The samples were part of a larger adolescent cohort (*n* = 4391) whose genotyping data were subjected to tests of quality control, including checks for pedigree, sex and Mendelian errors, and for ancestry using HapMap3 and GenomeEUTwin individuals as a reference panel. Five individuals were removed because of gender inconsistencies, and 28 individuals (14 twin pairs) because of non-European ancestry. Quality control filters, as previously described (Medland *et al*. 2009), ensured no samples had a call rate ≤0.95, and that all SNPs included in analyses had the following characteristics: call rate ≥ 0.95, minor allele frequency (MAF) ≥ 0.01 and Hardy-Weinberg equilibrium (HWE) test with *P* ≥ 1 × 10^−6^. The HapMap phase II CEU data [NCBI build 36 (UCSC hg18)] was used as the reference sample for imputation using MACH software. The SNPs with low imputation (*r*^2^ < 0.30) and low MAF (<0.01) were excluded. A total of 2 373 249 SNPs were examined.

#### ALSPAC sample

A broader sample of 9912 participants were genotyped using the Illumina HumanHap550 quad genome-wide SNP genotyping platform by 23andMe subcontracting the Wellcome Trust Sanger Institute, Cambridge, UK and the Laboratory Corporation of America, Burlington, NC, USA. Individuals were excluded from analyses on the basis of excessive or minimal heterozygosity, gender mismatch, individual missingness (>3%), cryptic relatedness as measured by identity by decent (genome-wide IBD > 10%) and sample duplication. Individuals were assessed for population stratification using multidimensional scaling modeling seeded with HapMap Phase II release 22 reference populations. Individuals of non-European ancestry were removed from further analysis. Individuals were imputed to HapMap Phase II (Build 36 release 22) using Markov Chain Haplotyping software [MACH v.1.0.16 (Li *et al*. 2010)], with removal of poorly imputed SNPs (*r*^2^ hat < 0.30).

### Statistical analyses

In the BATS, each reading and language measure was first residualized for the effects of sex, age, examiner and performance IQ. Genome-wide association analysis, implemented in Merlin, was conducted on standardized scores using a total test of association which corrected for sample relatedness (Chen & Abecasis 2007). In the ALSPAC sample, standardized residuals were derived for each trait controlling for the effects of sex, age and performance IQ and these were regressed on expected allelic dosage scores using the software package MACH2QTL (Li *et al*. 2009). A weighted inverse variance method in METAL (Willer *et al*. 2010) was used to meta-analyze the results. The reading and spelling factor from BATS was meta-analyzed with the word reading, non-word reading and spelling composite from ALSPAC (henceforth termed the ‘reading and spelling measure’). The Schonell from BATS was meta-analyzed with word reading from ALSPAC (henceforth termed the ‘word reading measure’), and the NWR measures from each cohort were meta-analyzed together.

A conventional genome-wide significance level of *P* < 5 × 10^−8^ was adopted (Dudbridge & Gusnanto 2008). Correcting for multiple testing of three variables, the level for genome-wide significance is *P* < 1.7 × 10^−8^. For reporting, significance at a suggestive level (*P* < 6.09 × 10^−6^ as proposed for the CEU SNP panel of markers adjusted for SNP non-independence) is used (Duggal *et al*. 2008). Empirical analyses indicate that uncorrected SNP associations of *P* < 1 × 10^−7^ typically constitute replicable associations (Panagiotou & Ioannidis 2012), although this may depend on factors such as adequate study power and trait heritability. For clarity, all *P*-values are reported uncorrected for multiple testing.

Gene-based tests were performed on the meta-analysis results using VEGAS, a program in which SNPs are assigned to genes, and their combined effect within a gene is tested taking into account SNP linkage disequilibrium (LD) (Liu *et al*. 2010). Roughly 18 000 autosomal genes were tested, annotated to positions on the UCSC Genome Browser (hg18 assembly) and including regulatory regions located ±50 kb of 5′ and 3′ untranslated regions. Bonferroni corrected significance was set at *P* < 2.8 × 10^−6^ based on a correction for the number of genes tested.

GWAS results were further used to check for replication of the previously associated candidate genes with dyslexia and SLI. This was also done within each cohort so that any future meta-analyses incorporating these results can test for the presence of sample heterogeneity thereby enabling the specification of random effects models.

## Results

Phenotypic distributions in both cohorts were approximately normal and extreme outliers were removed prior to analysis. The maximum included value was 5.42 SDs from the mean for the Schonell in the BATS and 3.03 SDs from the mean for the word reading score in the ALSPAC sample. All results of the GWAS analysis were tested for evidence of population stratification (see Q–Q plots presented in Fig. S1, Supporting Information), with *λ* values within an acceptable range of 1.016–1.026. There was some indication of SNP tests deviating from the null distribution for NWR.

### SNP-based GWAS results

The SNPs reaching a suggestive significance level are shown in Table1. The distribution of *P*-values across the 22 chromosomes is shown in Manhattan plots provided in Fig. S2 and the top 100 SNPs for each trait are provided in Table S1.

**Table 1:** Description and results (*β*, SE and *P*) of the top SNPs (*P* < 6.09 × 10^−6^) associated with reading and language measures

SNP	Ch	Position	A1	A2	AF	*P*	A1 *β*	SE	Gene	Other associations *P* < 0.05: *β*, SE, *P*
Reading and spelling (RS)										
rs4807927	19	1374199	A	G	0.94	1.32 × 10^−6^	0.207	0.043	*DAZAP1*	WR: 0.15 ± 0.03, 5.59 × 10^−6^; NWR: 0.15 ± 0.04, *P* = 0.0003
rs17135159	10	3398947	A	C	0.81, 0.80	2.40 × 10^−6^	0.107	0.023	*BC037918*	WR: 0.06 ± 0.02, *P* = 0.0009; NWR: 0.07 ± 0.02, *P* = 0.0006
rs479526	6	11726153	T	C	0.54, 0.55	2.71 × 10^−6^	0.093	0.020		WR: 0.05 ± 0.01, *P* = 0.0005
rs3213056	10	62218396	A	G	0.04, 0.03	3.34 × 10^−6^	−0.376	0.081	*CDC2*	WR: −0.23 ± 0.06, *P* = 7.65 × 10^−5^
rs10508253	10	3420951	T	G	0.86	3.77 × 10^−6^	0.121	0.026	*BC037918*	WR: 0.06 ± 0.02, *P* = 0.001; NWR: 0.10 ± 0.02, *P* = 9.75 × 10^−5^
rs13307587	7	13510254	A	G	0.66, 0.67	4.94 × 10^−6^	−0.088	0.019		WR: −0.06 ± 0.01, 7.09 × 10^−5^; NWR: −0.05 ± 0.02, *P* = 0.005
rs11666805	19	1376448	T	C	0.08, 0.07	5.71 × 10^−6^	−0.163	0.036	*DAZAP1*	WR: −0.12 ± 0.03, 1.70 × 10^−5^; NWR: −0.10 ± 0.03, *P* = 0.004
Word reading (WR)										
rs764255	16	72271184	T	C	0.63, 0.66	1.80 × 10^−7^	−0.077	0.015		RS: −0.07 ± 0.02, *P* = 0.0004
rs4839516	1	117000000	A	C	0.31	3.61 × 10^−7^	0.079	0.015		RS: 0.07 ± 0.02, *P* = 0.0004; NWR: 0.04 ± 0.02, *P* = 0.029
rs11158345	14	60964798	T	C	0.80, 0.82	3.29 × 10^−6^	0.084	0.018	*PRKCH*	RS: 0.10 ± 0.02, *P* = 1.32 × 10^−5^
rs1928007	13	69708224	A	G	0.91	4.77 × 10^−6^	−0.108	0.024		RS: −0.13 ± 0.03, *P* = 2.18 × 10^−5^; NWR: −0.07 ± 0.03, *P* = 0.015
rs11945798	4	12378330	T	C	0.96	4.94 × 10^−6^	−0.175	0.038		RS: −0.16 ± 0.05, *P* = 0.002
rs12050412	14	82447756	T	G	0.80	4.95 × 10^−6^	0.077	0.017		RS: 0.09 ± 0.02, *P* = 4.59 × 10^−5^
rs11577628	1	161000000	A	G	0.92	5.00 × 10^−6^	−0.123	0.027	*NOS1AP*	RS: −0.11 ± 0.04, *P* = 0.0016; NWR: −0.10 ± 0.04, *P* = 0.004
rs4807927	19	1374199	A	G	0.94	5.59 × 10^−6^	0.147	0.032	*DAZAP1*	RS: 0.21 ± 0.04, *P* = 1.32 × 10^−6^; NWR: .015 ± 0.04, *P* = 0.0003
rs1357978	7	13526746	A	C	0.30	5.84 × 10^−6^	0.070	0.016		RS: 0.09 ± 0.02, *P* = 5.46 × 10^−6^; NWR: 0.05 ± 0.02, *P* = 0.004
Non-word repetition (NWR)										
rs2192161	21	14603865	A	G	0.06	7.34 × 10^−8^	0.198	0.037	*ABCC13*	
rs2822560	21	14596503	T	C	0.05	8.73 × 10^−8^	0.216	0.040	*ABCC13*	
rs7187223	16	81015234	A	G	0.96	9.90 × 10^−8^	0.251	0.047		
rs2822609	21	14639665	A	T	0.96	4.64 × 10^−7^	−0.210	0.042		
rs12482528	21	14632164	T	C	0.07	6.04 × 10^−7^	0.189	0.037	*ABCC13*	
rs7202575	16	81005610	T	C	0.04	9.08 × 10^−7^	−0.227	0.046		
rs6954796	7	20053507	C	G	0.13	1.59 × 10^−6^	0.124	0.026		
rs2041660	7	20074148	T	C	0.13	3.30 × 10^−6^	0.124	0.027		
rs7777159	7	20051126	A	G	0.15	5.81 × 10^−6^	0.112	0.025		

A1, allele 1; A2, allele 2; AF, allele frequency (BATS, ALSPAC where unequal). Other significant SNPs that were in strong LD (*r*^2^ > 0.80) with the SNPs listed in the table: RS: rs17135159 with rs12415036, rs2182195, rs11251863 and rs11251868; rs12351590 with rs10960483, rs7025259, rs10960488 and rs791775; rs13307587 with rs12699504 and rs1357978; rs11666805 with rs3786983. WR: rs764255 with rs8059199, rs716270, rs716271, rs4243136, rs8048857, rs8056327, rs1480566, rs990258, rs4243137 and rs3956052; rs1928007 with rs7989551. NWR: rs2192161 with rs909257; rs2822560 with rs12329784, rs17002266, rs7283184 and rs2822584; rs7187223 with rs7196091, rs956315, rs7202694, rs2161694, rs1424049, rs6565017, rs1025066 and rs3909533; rs6954796 with rs1019248, rs4721865 and rs10246471; rs6954796 with rs2041664, rs2041663, rs7795980 and rs1005499.

The most significant association (*P* = 7.34 × 10^−8^) was observed between rs2192161—located in *ABCC13*—and NWR. The minor allele (frequency of 6%) was associated with increased reading skill. Two other SNPs in this gene were also suggestively significant. Figure S3 shows the results for the surrounding region of this SNP in detail including SNP LD and the location of nearby genes. The SNPs in *DAZAP1* were suggestively associated with the reading and spelling measure (*P* = 1.32 × 10^−6^; see Fig. S4) and the word reading measure (*P* = 5.59 × 10^−6^). Correlated traits that showed nominal significance are also highlighted; we were interested especially in whether reading and NWR SNP associations overlapped. Although 10 of the suggestively associated SNPs for the reading measures showed nominal association with NWR, none of the suggestively significant NWR associations were nominally associated with reading traits.

### Gene-based tests

Results for the 10 most significant genes according to VEGAS are shown in Table S2. All genes significant at a nominal level (*P* < 0.05) are shown in Table S3. Four genes (at two independent loci) associated with the reading and spelling measure reached the threshold for Bonferroni significance, including *CDC2L1*, *CDC2L2*, *LOC728661* and *RCAN3*. Four genes showing nominal significance for the reading and spelling measure also appeared in the top 10 for the word reading measure, including *CDC2L1*, *CDC2L2*, *LOC728661* and *RPS15*.

### Single SNP replication of dyslexia and SLI candidate genes from the literature

#### BATS

Our group has already published on candidate dyslexia genes *KIAA0319*, *DYX1C1* and *DCDC2* based on targeted genotyping of regions in a smaller dataset than is used here (Bates *et al*. 2010; Lind *et al*. 2010; Luciano *et al*. 2007, 2011). Since this time, additional subjects have entered the dataset, primarily recruited at age 12. Therefore, we re-examined the results for these SNP associations in the larger, but more age-diverse sample. It should be noted too that slight differences in the covariate adjustments were used between studies.

For *KIAA0319*, we had originally observed association with two SNPS; of these, rs2143340 was available in the current GWAS and was associated with the Schonell [major allele *β* =−0.06 (Standard Error (SE) 0.03), *P* = 0.02] but not the reading and spelling measure [major allele *β* =−0.09 (SE 0.06), *P* = 0.13]. For *DCDC2*, two SNPs had originally passed Bonferroni significance for association with CORE measures, but only one of these was available in the GWAS (rs1419228) and it fell short of significance with the CORE reading and spelling measure [major allele *β* = 0.106 (SE 0.06), *P* = 0.08].

We checked evidence of association between NWR and SNPs previously associated with SLI, having previously published on *ROBO1* variants from the current GWAS (Bates *et al*. 2011). The results for *CMIP* and *ATP2C2* genes (Newbury *et al*. 2009) were as follows: in *CMIP*: rs12927866, major (C) allele *β* = 0.071 (SE 0.05), *P* = 0.13; rs4265801, major (G) allele *β* =−0.062 (SE 0.05), *P* = 0.18; rs16955705, major (A) allele *β* = 0.087 (SE 0.05), *P* = 0.06, and three SNPs in *ATP2C2*: rs16973771, major (T) allele *β* = 0.023 (SE 0.05), *P* = 0.64; rs2875891: major (C) allele *β* = 0.042 (SE 0.05), *P* = 0.40; rs8045507, major (G) allele *β* = 0.027 (SE 0.05), *P* = 0.58. Nine SNPS in *CNTNAP2* reached corrected significance in the Vernes *et al*. (2008) study of nonsense word repetition in children with SLI and their non-affected siblings. Six of these were available in our GWAS: rs851715, major (T) allele *β* = 0.046 (SE 0.05), *P* = 0.35; rs10246256, major (T) allele *β* = 0.05 (SE 0.05), *P* = 0.32; rs2710102, major (G) allele *β* = 0.038 (SE 0.05), *P* = 0.41; rs759178, major (C) allele *β* = 0.038 (SE 0.05), *P* = 0.41; rs17236239, major (A) allele *β* = 0.03 (SE 0.05), *P* = 0.52 and rs2710117, major (A) allele *β* = 0.058 (SE 0.05), *P* = 0.21. Finally, of the 11 available SNPs in LD with a haplotype found to be significantly associated with SLI in an isolated Chilean population (three of which were located in *CNTNAP2*) (Villanueva *et al*. 2011) none showed association.

#### ALSPAC sample

Targeted SNP association has previously been reported in ALSPAC for variants in *MRPL19*/*C2ORF3*, *DCDC2*, *KIAA0319*, *CMIP* and *ATP2C2*: only rs2143340 (in *KIAA0319*) showed Bonferroni-corrected association with reading ability (Scerri *et al*. 2011). Because these analyses excluded reading traits at age 9 and were conducted in a smaller sample (*N* ∼ 3700) that had filtered out 1036 individuals with non-white ethnicity, low performance IQ (<85) and likely presence of autistic traits we report the result for rs2143340 here. In the current GWAS dataset, association with rs2143340 was marginally significant (i.e. ∼*P* = 0.05) with the reading and spelling measure [minor allele *β* =−0.052 (SE 0.03), *P* = 0.06] and not associated with word reading [minor allele *β* =−0.008 (SE 0.03), *P* = 0.77]. For *DYX1C1* marker rs17819126, the reading and spelling measure showed no association [major allele *β* =−0.002 (SE 0.04), *P* = 0.96] nor did the word reading measure [major allele *β* = 0.007 (SE 0.04), *P* = 0.86]. In the BATS, this marker was only associated with non-word reading (Bates *et al*. 2010)

For NWR, SNPs in *CMIP* and *ATP2C2* have been previously tested for association in ALSPAC and had not been found to be significant (Newbury *et al*. 2009). Two SNPs (rs6803202 and rs4535189) in high LD in *ROBO1* and associated with NWR in the BATS sample (Bates *et al*. 2011) were not significantly associated with NWR in ALSPAC (*P* = 0.88). The SNPS in *CNTNAP2* previously reported as associated with nonsense word repetition (Vernes *et al*. 2008) showed the following results: rs851715, major (T) allele *β* = 0.012 (SE 0.02), *P* = 0.56; rs10246256, major (T) allele *β* = 0.013 (SE 0.02), *P* = 0.55; rs2710102, major (G) allele *β* =−0.028 (SE 0.02), *P* = 0.15; rs759178, major (C) allele *β* =−0.028 (SE 0.02), *P* = 0.14; rs17236239, major (A) allele *β* = 0.037 (SE 0.02), *P* = 0.06 and rs2710117, major (A) allele *β* = 0.01 (SE 0.02), *P* = 0.70. Of the 11 SNPs in LD with a haplotype reported as associated with SLI (Villanueva *et al*. 2011) we found association approaching significance with one: rs1371463 [minor allele β =−0.04 (SE 0.02), *P* = 0.08] in *SVOPL*.

#### Meta-analysis sample

Meta-analysis results of the replication SNPs detailed above showed nominally significant results for: rs17236239 (in C*NTNAP2*) where major (A) allele *β* = 0.04 (SE 0.02), *P* = 0.05 and for rs1371463 (in *SVOPL*) where major (C) allele *β* = 0.04 (SE 0.02), *P* = 0.03. All other results were non-significant.

## Discussion

Twenty-five independent SNPs were shown to be suggestively associated with three measures of reading and language in cohorts of British children and primarily adolescent Australians. The most significant association (*P* = 7.34 × 10^−8^) was observed between rs2192161—located in the *ABCC13* [ATP-binding cassette, subfamily C (CFTR/MRP), member 13] pseudogene on 21q11.2—and NWR, the measure of language. Given that there was no nominal association of this SNP to the reading measures, this might be an SLI-specific locus. It is not located near previously reported dyslexia loci and given its low MAF (6%) this finding might represent type 1 error, but if not, this variant confers a large effect, explaining 3.9% of the variance. *ABCC13* is an ABC gene that differs functionally between monkeys and apes. The gene codes for a functional protein in the rhesus macaque, where it plays a role in transport of ATP across membranes (Annilo & Dean 2004). In humans, it is a pseudogene with no known role in transporting activity but instead encodes a truncated protein that is expressed in fetal human liver, similar to *ABCC2* (Zhou *et al*. 2008).

All the suggestively significant SNP associations for the reading and spelling measure showed nominal significance for the word reading measure and vice versa. One SNP, rs4807927 [located in DAZ-associated protein 1 (*DAZAP1*)], was associated with both the reading and spelling measure and the word reading measure at a suggestive significance level, so it may represent a more robust finding than the others given that these traits are moderately correlated. Most of these SNPs were also nominally associated with the language measure. The overlap in SNP findings across traits might suggest their effect on general reading (and even language) processes. A multivariate analysis of more distinct individual reading component measures is needed to confirm the magnitude of any shared effects. If the SNP effect is at the ‘general processes’ level, then such analysis might even increase statistical power (O’Reilly *et al*. 2012).

Four of the gene-based tests reached Bonferroni-corrected significance for the reading and spelling measure, including *CDC2L1* (Cell Division Cycle 2-Like 1), *CDC2L2* (Cell Division Cycle 2-Like 2), *LOC728661* and *RCAN3* (RCAN family member 3) located on chromosome 1. The first three genes represented one locus because their chromosomal region overlapped, but *RCAN3* was an independent locus. Four genes appeared in the top 10 most significant results for both the reading and spelling measure and the word reading measure: *CDC2L1*, *CDC2L2*, *LOC728661* and *RPS15* (Ribosomal Protein S15). These genes have not previously been reported in relation to reading traits and their biological relevance as candidate genes is unclear.

Previously implicated SNPs/genes associated with reading or SLI showed somewhat inconsistent, albeit potentially informative, results across our two cohorts. As previously reported by Luciano *et al*. (2007), rs2143340 in *KIAA0314* was associated with word reading in BATS but was only marginally significant in ALSPAC. In a subsample of ALSPAC, which excluded non-white, very low IQ and potentially autistic children this SNP was, however, found to be significant (Scerri *et al*. 2011). Our finding suggests, then, that background population structure as discussed by Paracchini *et al*. (2008) can be a barrier to replication for genetic association. The exclusion of individuals with comorbidities (like autism and Attention Deficit Hyperactivity Disorder) may also be advisable, reducing non-reading specific variation in reading ability attributable instead to comorbid disorders. Of course, for a complete understanding of genetic influences on reading ability, it will be important also to understand and incorporate these alternate influences on reading ability.

Skiba *et al*. (2011) have discussed at length the problems that phenotype heterogeneity present for replication studies of reading ability/dyslexia. The use of comorbid reading and language impaired samples has been shown to produce a particularly sensitive genetic design, perhaps reflecting severe affection (e.g. see Scerri *et al*. 2011). By contrast, if genes have specific functionality, such comorbidity may diffuse genetic signals. In the present case, for instance, *DYX1C1* SNP, rs1781926, was not associated with the reading measures in the ALSPAC. However, this SNP was associated in BATS with non-word reading (Bates *et al*. 2010). This may be interpreted as an example of gene specificity, in this case for phonological decoding. For NWR, replication results approached significance for SNPs in *CMIP* (in BATS but not ALSPAC) and in *CNTNAP2* and *SVOPL* (for both genes in ALSPAC but not BATS). Again, differences between cohorts due to ethnic composition and age might explain these discrepancies. Because these replication results were not the focus of our investigation, we only presented results for the most relevant previous SNP associations; obviously, a more thorough replication analysis should focus on all SNPs in the gene with adjustments made for within-gene SNP LD.

The cohorts in this study differed significantly in age: in the Australian sample, the mean age was 17.9 years, a period when reading acquisition is largely stable; in the British sample, aged 9 years, reading skill is still actively being acquired, and environmental and developmental timing differences may play a larger role in the phenotype. It may even be the case that different genetic substrates are involved in this earlier phase of reading acquisition. Our GWAS results therefore apply to gene effects on reading ability, which are present across both childhood and adolescence and into young adulthood. Previous research on the increase of heritability across development, and the continuity of genetic influences suggests increasing heritability or genetic penetrance with age, and has supported both substantial genetic continuity and significant genetic innovation. For instance, a longitudinal adoption study (Wadsworth *et al*. 2001) showed that between the ages of 7 and 16 years common genetic effects explain 88% of the stability in reading performance. Moreover, no new genetic effects emerged at age 12 or 16, suggesting that the genetic effects at age 7 were simply amplified later on. However, a much larger longitudinal twin study of teacher-assessed reading achievement across the ages of 7, 9 and 10 years showed considerable genetic continuity (68% and 77% of stability) and also evidence for novel genetic contributions at both the latter two ages (Harlaar *et al*. 2007). Clearly, more GWAS samples are needed to obtain the increased power required to investigate age-specific genome-wide effects on reading ability. The loci identified in this study (including the top 100 SNP associations and gene-based tests) will serve as an important repository for future GWAS of reading and language abilities and disorders to confirm overlap with their own findings.

## Supporting Information

Table S1 GWA results for the 100 most significant associations for measures of reading and spelling, word reading and non-word repetition.

Table S2 Top 10 most significant genes for reading and language traits as evaluated by VEGAS. Significant genes appear in bold and genes associated with multiple traits are superscripted.

**Table S3:** Nominally significant (P <0.05) results for genebased tests of association with reading and spelling, word reading and non-word repetition.

Figure S1 Q–Q plots of the GWAS association metaanalysis results for reading and spelling, word reading and non-word repetition.

Figure S2 Manhattan plots depicting the GWAS association meta-analysis results for reading and spelling, word reading and non-word repetition.

Figure S3 Annotation for NWR association in region surrounding rs2192161. Linkage disequilibrium is represented by r2. No non-synonymous coding SNPs can be observed.

Figure S4 Annotation for the reading and spelling association in the region surrounding rs4807927. Linkage disequilibrium is represented by r2. Non-synonymous () and coding () SNPs can be observed.
