# Supplementary material for: A genome-wide association study for reading and language abilities in two population cohorts
Source: Genes Brain Behav. 2013 Jun 20;12(6):645–52. doi: 10.1111/gbb.12053 (PMC3908370; doi:10.1111/gbb.12053)
Supplement: Table S2 — Top 10 most significant genes for reading and language traits as evaluated by VEGAS. Significant genes appear in bold and genes associated with multiple traits are superscripted. [file gbb0012-0645-sd2.doc]

**Supplementary Table 2.** Top 10 Most Significant Genes for Reading and Language Traits as Evaluated by VEGAS. Significant Genes Appear in Bold and Genes Associated with Multiple Traits are Superscripted.

| Gene | Ch | Start Position | Stop Position | No. SNPS in Gene | p-value |
| --- | --- | --- | --- | --- | --- |
| **Reading and Spelling** |  |  |  |  |  |
| ***CDC2L1****^1^* | 1 | 1560962 | 1645635 | 3 | 1.00 x10^-6^ |
| ***CDC2L2****^2^* | 1 | 1624029 | 1645651 | 3 | 2.00 x10^-5^ |
| ***LOC728661****^3^* | 1 | 1582801 | 1614103 | 3 | 2.00 x10^-5^ |
| ***RCAN3*** | 1 | 24701973 | 24735014 | 87 | 2.70 x10^-5^ |
| *C1orf130* | 1 | 24755188 | 24808403 | 84 | 6.10x10^-5^ |
| *RPS15^4^* | 19 | 1389362 | 1391492 | 38 | 0.000171 |
| *MEF2C* | 5 | 88051921 | 88214780 | 159 | 0.000188 |
| *CACNB3* | 12 | 47498778 | 47508991 | 60 | 0.000243 |
| *TIPRL* | 1 | 1.66E+08 | 1.66E+08 | 72 | 0.000278 |
| *FAM165B* | 21 | 34669618 | 34683322 | 80 | 0.000324 |
| **Word Reading** |  |  |  |  |  |
| *PIAS4* | 19 | 3958748 | 3989067 | 53 | 5.10x10^-5^ |
| *EEF2* | 19 | 3927053 | 3936461 | 45 | 0.000102 |
| *ZNF707* | 8 | 1.45E+08 | 1.45E+08 | 37 | 0.000119 |
| *SPAG6* | 10 | 22674404 | 22746545 | 91 | 0.000135 |
| *TUFT1* | 1 | 1.5E+08 | 1.5E+08 | 76 | 0.00015 |
| *DIRC1* | 2 | 1.89E+08 | 1.89E+08 | 166 | 0.000191 |
| *CDC2L1^1^* | 1 | 1560962 | 1645635 | 3 | 0.000325 |
| *RPS15^4^* | 19 | 1389362 | 1391492 | 38 | 0.000326 |
| *CDC2L2^2^* | 1 | 1624029 | 1645651 | 3 | 0.000329 |
| *LOC728661^3^* | 1 | 1582801 | 1614103 | 3 | 0.000334 |
| **Non-word Repetition** |  |  |  |  |  |
| *ADAMTS8* | 11 | 129780027 | 129803749 | 83 | 0.000111 |
| *APOC2* | 19 | 50141082 | 50144658 | 66 | 0.000154 |
| *H3F3A* | 1 | 224317043 | 224326326 | 48 | 0.000162 |
| *APOC4* | 19 | 50137334 | 50140591 | 69 | 0.000164 |
| *CLPTM1* | 19 | 50150477 | 50188439 | 73 | 0.00017 |
| *RELB* | 19 | 50196551 | 50233292 | 61 | 0.000255 |
| *UBL3* | 13 | 29236541 | 29322160 | 207 | 0.000297 |
| *ADPRH* | 3 | 120781212 | 120791482 | 109 | 0.000333 |
| *ADAMTS15* | 11 | 129824078 | 129848926 | 90 | 0.000353 |
| *TMEM55A* | 8 | 92075674 | 92122224 | 85 | 0.000425 |
