# Supplementary figures and images for: A genome-wide association study for reading and language abilities in two population cohorts
Source: Genes Brain Behav. 2013 Jun 20;12(6):645–52. doi: 10.1111/gbb.12053 (PMC3908370; doi:10.1111/gbb.12053)

## Slide 1
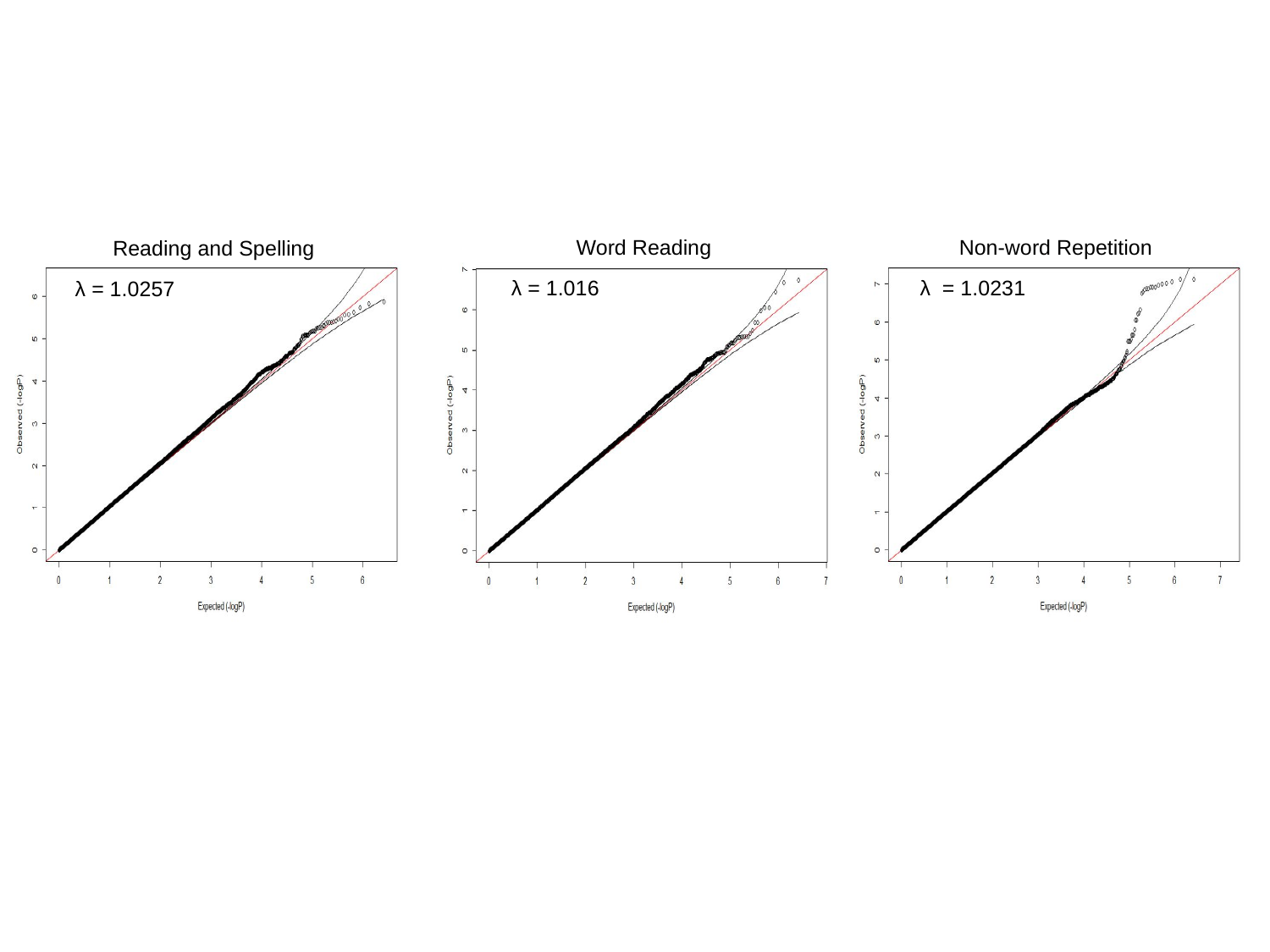

Non-word Repetition
Word Reading
Reading and Spelling
λ = 1.016
λ = 1.0231
λ = 1.0257

Supplement: Figure S1 — Q–Q plots of the GWAS association metaanalysis results for reading and spelling, word reading and non-word repetition. [file gbb0012-0645-sd4.ppt]

## Slide 1
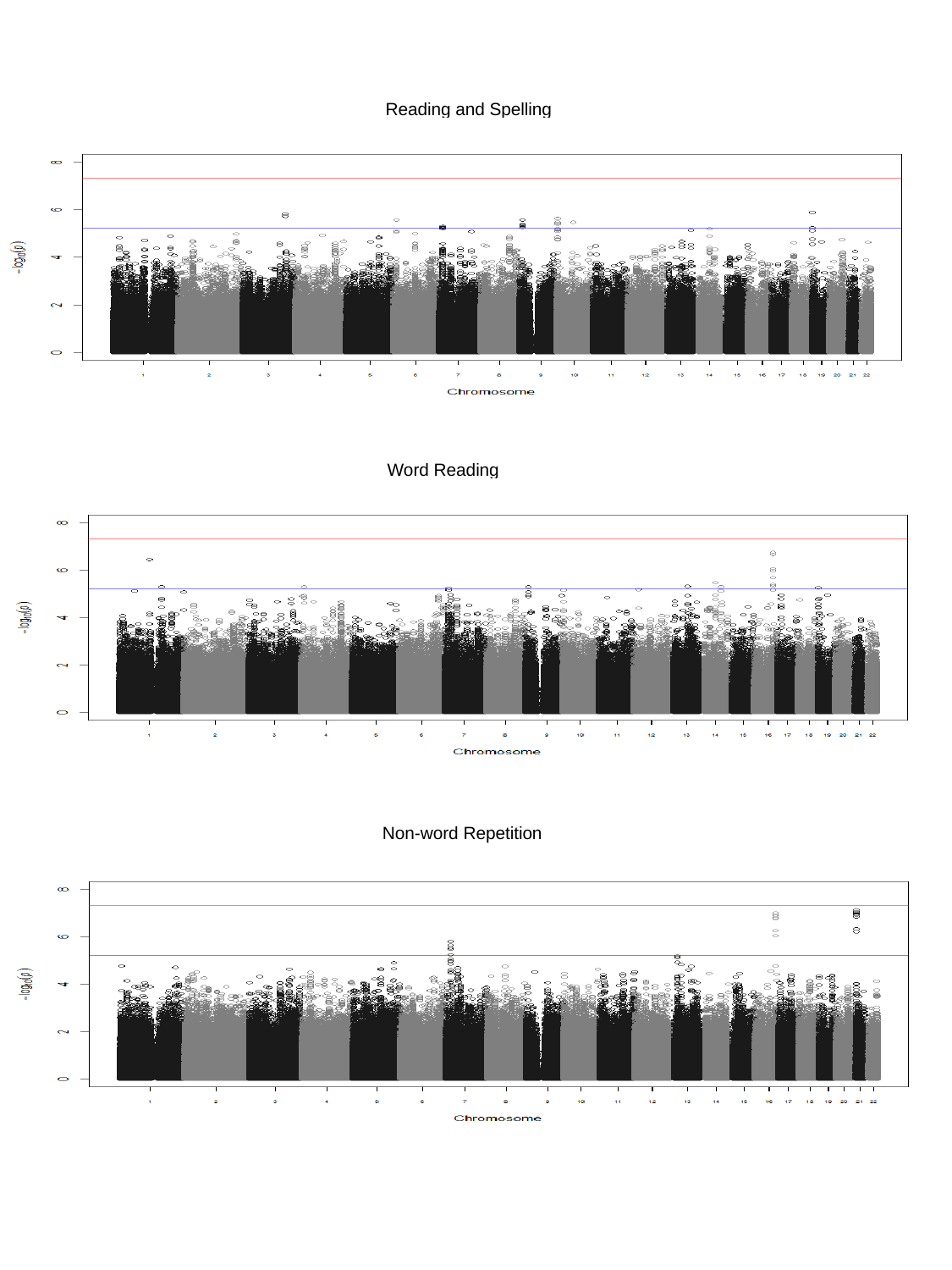

Reading and Spelling
Word Reading
Non-word Repetition

Supplement: Figure S2 — Manhattan plots depicting the GWAS association meta-analysis results for reading and spelling, word reading and non-word repetition. [file gbb0012-0645-sd5.ppt]
