# Supplementary material for: A genome-wide association study for reading and language abilities in two population cohorts
Source: Genes Brain Behav. 2013 Jun 20;12(6):645–52. doi: 10.1111/gbb.12053 (PMC3908370; doi:10.1111/gbb.12053)
Supplement: Figure S3 — Annotation for NWR association in region surrounding rs2192161. Linkage disequilibrium is represented by r2. No non-synonymous coding SNPs can be observed. [file gbb0012-0645-sd6.ppt]

## Slide 1
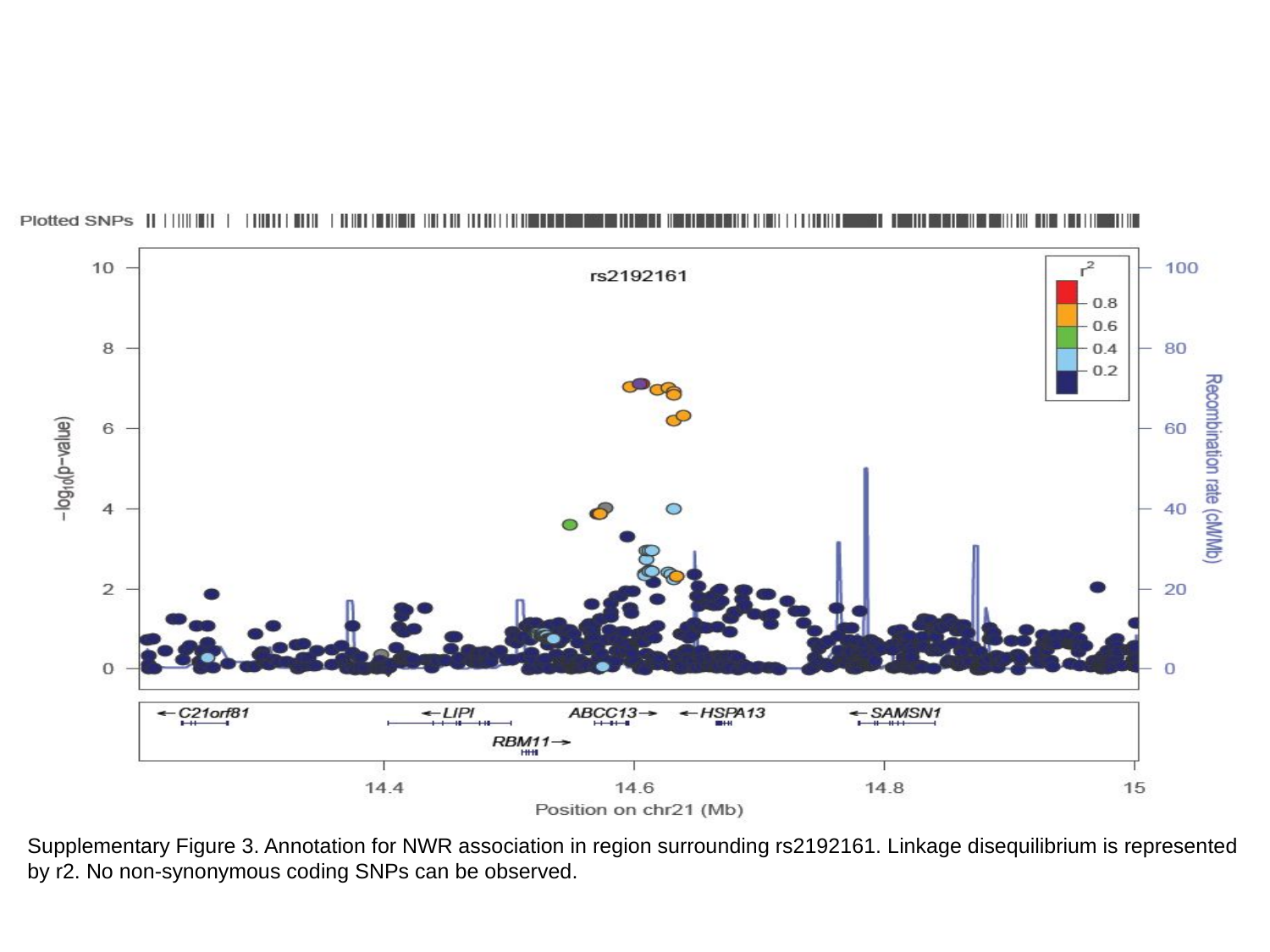

Supplementary Figure 3. Annotation for NWR association in region surrounding rs2192161. Linkage disequilibrium is represented by r2. No non-synonymous coding SNPs can be observed.
