# Supplementary material for: A genome-wide association study for reading and language abilities in two population cohorts
Source: Genes Brain Behav. 2013 Jun 20;12(6):645–52. doi: 10.1111/gbb.12053 (PMC3908370; doi:10.1111/gbb.12053)
Supplement: Figure S4 — Annotation for the reading and spelling association in the region surrounding rs4807927. Linkage disequilibrium is represented by r2. Non-synonymous () and coding () SNPs can be observed. [file gbb0012-0645-sd7.ppt]

## Slide 1
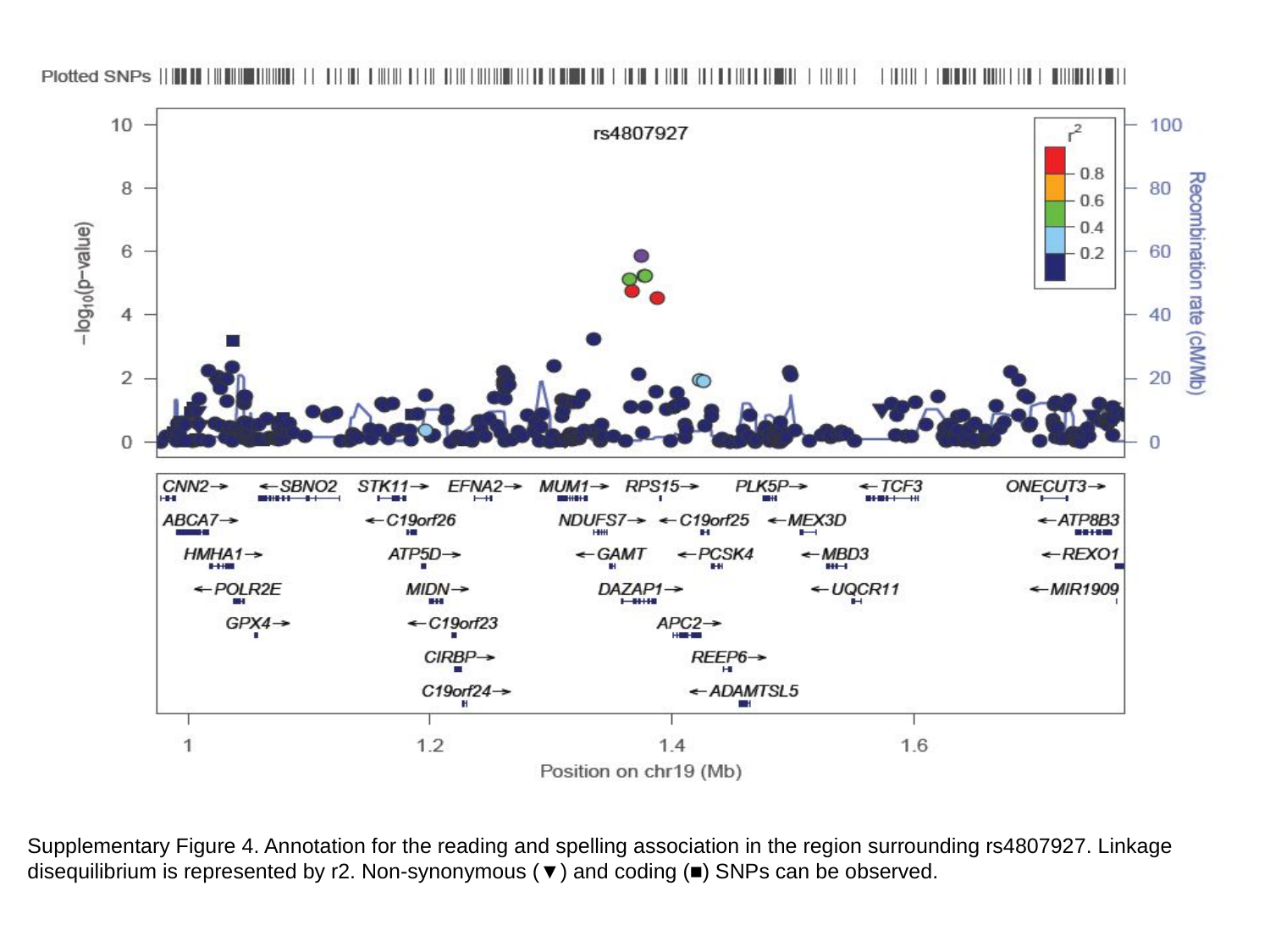

Supplementary Figure 4. Annotation for the reading and spelling association in the region surrounding rs4807927. Linkage disequilibrium is represented by r2. Non-synonymous (▼) and coding (■) SNPs can be observed.
